# Assessment of the Performance of Cationic Cellulose Derivatives as Calcium Carbonate Flocculant for Papermaking

**DOI:** 10.3390/polym14163309

**Published:** 2022-08-14

**Authors:** Jorge F. S. Pedrosa, Luís Alves, Carlos P. Neto, Maria G. Rasteiro, Paulo J. T. Ferreira

**Affiliations:** 1 University of Coimbra, CIEPQPF, Department of Chemical Engineering, Pólo II, R. Sílvio Lima, 3030-790 Coimbra, Portugal; 2 Forest and Paper Research Institute (RAIZ), R. José Estevão, Eixo, 3800-783 Aveiro, Portugal

**Keywords:** calcium carbonate, cationic cellulose, fibrillated cellulose, flocculation, laser diffraction spectrometry, quaternary ammonium

## Abstract

Cationic polyacrylamides (CPAMs) are usually used as filler retention agents in papermaking formulations. However, increasing environmental restrictions and their non-renewable origin have driven research into bio-based alternatives. In this context, cationic lignocellulosic derivatives have been attracting considerable research interest as a potential substitute. In this work, distinct cationic celluloses with degrees of substitution of between 0.02 and 1.06 and with distinct morphological properties were synthesized via the cationization of bleached eucalyptus kraft pulp, using a direct cationization with (3-chloro-2-hydroxypropyl) trimethylammonium chloride (CHPTAC) or a two-step cationization, where the cellulose was first oxidized to form dialdehyde cellulose and was then made to react with Girard’s reagent T (GT). Fibrillated samples were produced by subjecting some samples to a high-pressure homogenization treatment. The obtained samples were evaluated regarding their potential to flocculate and retain precipitated calcium carbonate (PCC), and their performance was compared to that of a commercial CPAM. The cationic fibrillated celluloses, with a degree of substitution of ca. 0.13–0.16, exhibited the highest flocculation performance of all the cationic celluloses and were able to increase the filler retention from 43% (with no retention agent) to ca. 61–62% (with the addition of 20 mg/g of PCC). Although it was not possible to achieve the performance of CPAM (filler retention of 73% with an addition of 1 mg/g of PCC), the results demonstrated the potential of cationic cellulose derivatives for use as bio-based retention agents.

## 1. Introduction

In certain paper product formulations, a portion of the cellulose fibers is often partially replaced by mineral fillers (from now on, these will just be referred to as fillers), such as kaolin clay, talc, natural ground calcium carbonate (GCC) or synthetic precipitated calcium carbonate (PCC) [1].

Fillers, initially applied due to their lower price compared to cellulose fibers, are also used to improve a range of properties [2]. The use of fillers can improve the brightness and opacity of the paper, decrease the surface roughness (especially after calendaring), enhance the printing quality, improve the formation of the paper matrix by filling the voids between the fibers and also increase the dimensional stability of the paper, as they tend to remain inert when wetted, unlike cellulose fibers that can swell and retract [3].

These fillers present a typical particle size smaller than 4 µm [2], being much smaller than the mesh size of the screen at the forming section of the paper machine. In fact, the screens with a mesh size ranging between 40 and 100 mesh (openings with a diameter in the 150 to 400 µm range) [4], although small enough to retain almost the totality of the cellulose fibers (with a length of around 2–5 mm for softwood and 0.3–1.5 mm for hardwoods) [5], are not sufficiently small to retain the filler particles, resulting in material losses.

Although some fillers can present a slightly cationic surface, most of the commercial fillers present a negative surface charge, due to the use of dispersants [6]. With the cellulose fibers also presenting a net negative charge (mainly as a result of the ionization of carboxylic and sometimes sulfonic acidic groups that are introduced onto the fiber surface during the chemical pulping and bleaching steps) [7], cationic synthetic polymers are applied in papermaking formulations as retention agents to minimize the losses of fillers and/or small fibrous fragments and to take advantage of the natural affinity between oppositely charged particles.

Cationic polyacrylamides (CPAMs) with high molecular weight (Mw), medium to high charge density (CD) and linear or branched chains are frequently used as retention agents [8]. Although PAMs are considered non-toxic in their native polymeric form, their non-renewable origin and the existence in the final product of some residual acrylamide monomers [9] pose environmental and human health concerns since they exhibit a high degree of neurotoxicity [10]. These concerns, together with increasing environmental restrictions, pave the way for the development of new natural-based alternatives to be used as retention systems.

In the paper industry, the use of cellulose as a renewable resource that can serve as the backbone for the development of new additives, such as retention additives, appears to offer a logical alternative. Over the last few decades, lignocellulosic materials have been functionalized and/or deconstructed into their hierarchical sub-structures to produce new cellulose-based products, such as micro- and nanofibrillated celluloses (MNFCs). Purer forms of cellulose can be synthesized by some bacterial species (referred to as bacterial cellulose) and are typically explored for biomedical applications [11]. Some of these new products have already been tested in papermaking formulations and have shown their positive potential as filler retention agents [12,13].

From the published literature, it is possible to observe that nonionic or anionic cellulose-based products are far more common than their cationic counterparts, as observed by other authors [14].

In most recent years, the graft of cationic groups (typically, quaternary ammonium) into cellulosic materials, including lignocellulosic residues, has been explored as a possible alternative for the production of bio-based retention agents for the paper industry. However, the studies published are still relatively scarce and, in some cases, inconclusive [15,16,17,18,19].

Diab et al. [17] studied the use of cationic MFC in single and dual systems with bentonite for the retention of GCC in sheets produced with softwood and bagasse pulps. No improvements were observed in the filler retention, the results being attributed to the low degree of cationization (0.27 mmol/g). On the contrary, Li et al. [19] reported improvements in GCC retention by modifying the filler surface with soluble cationic cellulose, with a degree of substitution (DS) of 0.52.

For cellulose cationization, two main strategies are typically described in the literature. The first, the simplest and most applied strategy, comprises the direct reaction between cellulose and the reagent epoxy-propyltrimethylammonium chloride (EPTAC) [20,21,22]. Pedrosa et al. [23] instead used the precursor (3-chloro-2-hydroxypropyl) trimethylammonium chloride (CHPTAC) and converted it to the more reactive form EPTAC, via a reaction with sodium hydroxide.

The second cationization method is based on a two-step reaction in which cellulose is initially oxidized with sodium metaperiodate (NaIO4), causing the conversion of the two vicinal hydroxyl groups at the C2 and C3 positions into aldehyde groups and the subsequent cleavage of the C2–C3 bond. The resulting dialdehyde cellulose (DAC) can then react with Girard’s reagent T to form a stable imine structure that includes the quaternary ammonium groups [23,24,25,26].

The polymeric retention additives work through a process of chemical flocculation by the destabilization of the particles (fillers) in suspension. Three distinct mechanisms are typically referred to as being responsible for particle aggregation. Charge neutralization (or coagulation) occurs when a salt or low Mw polymer neutralizes the surface charge of the suspended particles, reducing the repulsive forces that separate them. Patching occurs by the creation of patches on the surface of the suspended particles of opposite charge, creating a few oppositely charged zones that can interact with the non-patched areas of other particles, resulting in flocculation via electrostatic forces. This mechanism occurs preferentially by using low or medium Mw and high-charge polymers. Finally, in the bridging mechanism, polymers with high Mw and low charge density are preferably used. The polymers adsorb on the surface of the particles in an extended conformation, forming long tails and loops that can extend beyond the electrical double layer of the particles. The extended branches of the polymer can then adsorb into other particles, forming polymeric bridges between them [5,7,27,28,29].

The coagulation-flocculation mechanisms and the performance of the retention agents are highly dependent on the polymer type, the suspended particles to be flocculated and the conditions of the medium [30], this being the predominant mechanism that is mainly dictated by the Mw and CD of the polymer [31]. For example, Aguado et al. [15] tested the use of water-soluble cationic derivatives obtained from bleached *Eucalyptus globulus* kraft pulp (BEKP) to flocculate distinct fillers (kaolinite, GCC and PCC). With the dosages tested (10 or 20 mg/g), only the derivative with the highest degree of polymerization (DP) and CD (1703 and 5 mmol/g, respectively) showed promising results, but this was only for kaolin flocculation.

When developing new retention additives, it is critical to understand the dominant mechanisms, the flocculation kinetics and the overall structure of the formed flocs. For papermaking tests, the flocculation performance of an additive is typically assessed via hydrodynamic techniques (the dynamic drainage jar test), by evaluating the drainage times and filler retention, and also by monitoring the zeta-potential (ZP) of the suspensions (which is most important for those mechanisms based on electrostatic interactions) [31,32].

More sophisticated alternative techniques based on light scattering, such as focused beam reflectance microscopy (FBRM) [33,34] and laser diffraction spectrometry (LDS) [31,32], have also been used, especially for their capability for real-time monitoring of the floc sizes. LDS not only allows the determination of the floc size distribution at each moment but also permits the extraction of information about the fractal dimension of the flocs that can be related to the compactness of the flocs formed [31]. LDS has already proven to be a very useful technique by which to assess the flocculation performance of polymers in real time, having already been used for the screening of synthetic polyelectrolytes [31,32,35], anionic MNFCs [12,36] and cationic cellulose polyelectrolytes [15,37,38].

**Figure 1:**
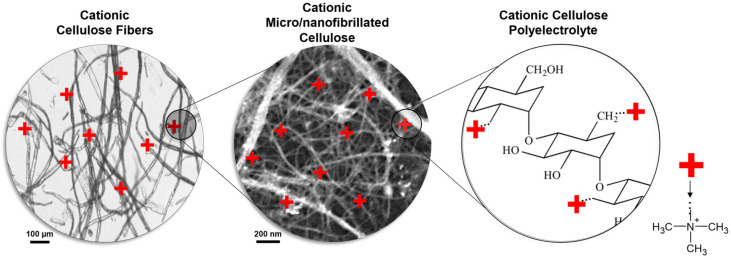
Schematic representation of cationic celluloses with distinct morphologies.

In the present work, two cationization methods were applied to produce several cationic celluloses (CCs) with distinct DS and morphological properties (fibers, micro/nanofibrillated celluloses and polyelectrolytes) (Figure 1). LDS was used to investigate the effect of the cationic cellulose characteristics, flocculant dosage and contact time on the flocculation of one of the most frequently used fillers in papermaking (PCC).

The performances were compared against a commercial CPAM and the initial non-functionalized cellulose fibers. The samples presenting the best flocculation performance in the LDS tests were incorporated into pulp and filler formulations and then analyzed in a dynamic drainage analyzer (DDA) to quantify the effects on drainability and the most effective filler retention capabilities.

Due to the importance of filler retention in papermaking and the constantly increasing health and environmental concerns/restrictions, the present study aims to shed some light on the potential use of CCs as a retention agent and as a possible CPAM substitute.

## 2. Materials and Methods

### 2.1. Materials

The cationic celluloses were produced from industrial unrefined and never-dried BEKP (80–85 wt % cellulose, 14–19 wt % xylan, 0.3 wt % lignin and 0.4 wt % extractives) [23]. A commercial linear CPAM (from BASF, Ludwigshafen, Germany), with a Mw of 3.7 × 10^6^ Da and a CD of 1.1 mmol/g, was used as the reference retention agent. An industrial scalenohedral form of PCC with a median diameter (d_50_) of 4.8 µm, determined by LDS, in a Mastersizer 2000 (Malvern Inst., Worcester City, UK), and a ZP of +9 mV (at pH 10), measured in a water suspension using electrophoretic light scattering (ELS–Zetasizer NanoZS, Malvern Inst., Malvern, Worcester City, UK), was used as filler in the flocculation tests.

All chemicals employed in the cationization of cellulose were used as received, without further purification. The (3-chloro-2-hydroxypropyl) trimethylammonium chloride (CHPTAC) 60 wt % aqueous solution, sodium periodate (SP) and (carboxymethyl)trimethylammonium chloride hydrazide (Girard’s reagent T–GT) were obtained from Sigma-Aldrich (Schnelldorf, Germany). Sodium hydroxide pellets and glacial acetic acid (AA) were purchased from VWR (Carnaxide, Portugal) and isopropanol (IPA) from Labsolve (Lisbon, Portugal). Distilled water was used throughout the work.

### 2.2. Cellulose Cationization

The cationic celluloses were produced, following two previously reported procedures [23], and under the conditions presented in Table 1.

Briefly, for the direct cationization with CHPTAC, BEKP was subjected to an activation step under alkaline conditions; then, a certain quantity of CHPTAC was added to the alkaline suspension. The cationic fibers were vacuum-filtered and thoroughly washed with distilled water.

For the two-step cationization, BEKP was first subjected to an oxidation reaction with sodium periodate and was consequently converted into DAC. The obtained DACs, with distinct degrees of oxidation, were washed with water and characterized for their aldehyde content, as described elsewhere [39]. The DACs were dispersed in acidified water and a certain molar ratio of GT/aldehyde was added. The cationic celluloses were vacuum filtered and thoroughly washed with a mixture of isopropanol/water (9:1 *v/v*), with the exception of sample GT1.15_P. This sample, due to its high degree of substitution and consequent high solubility, was washed in successive centrifugation cycles [37].

The produced cationic celluloses were labeled according to the cationizing agent used, with the acronyms CH or GT, followed by the degree of substitution.

Some of the cationic celluloses were further processed to produce cationic MNFCs. For that purpose, they were subjected to a pass at 500 bar and a second pass at 700 bar in a high-pressure homogenizer (HPH; GEA Niro Soavi, model: Panther 115 NS3006L). These samples were also labeled with the letter F (F stands for fibrillated). More details about the preparation of these samples are available elsewhere [23]. The samples that resulted in fully soluble materials, due to their high DS or the combination of cationization with HPH, were labeled with the letter P (P stands for polyelectrolyte).

### 2.3. Cationic Cellulose Characterization

The CCs were characterized by elemental analysis, potentiometric titration, ZP measurements, FTIR-ATR and optical microscopy [23]. Elemental analysis was performed in an EA 1108 CHNS-O analyzer from Fisons (Italy) to quantify the nitrogen content of the samples, which was used to calculate the corresponding DS of the quaternary ammonium groups. The CD was assessed using potentiometric titration. In a typical titration, a cationic cellulose suspension is adjusted to pH 11 with a NaOH aqueous solution and then titrated with 0.01 M HCl until the inflection point of the pH versus HCl volume curve is reached. The ZP of the diluted CCs suspensions (ca. 0.1 wt %) was determined via ELS in a Zetasizer NanoZS device from Malvern Instruments. The FTIR-ATR spectra were obtained on a Bruker Tensor 27 spectrometer, using 128 scans and a resolution of 4 cm^−1^, in the range of 650–4000 cm^−1^. Polarized light optical microscopy images were acquired using an Olympus BH-2 KPA microscope from the Olympus Optical Co., Ltd., equipped with an Olympus ColorView III high-resolution CCD color camera.

The cationic MNFCs were further characterized in terms of the yield of fibrillation (YF) and soluble fraction (SF). The YF was determined via centrifugation of 40 mL of CCs aqueous suspensions (0.2 wt %) at 9000 rpm for 30 min, using a Universal 32 Hettich centrifuge. The weight percentage of CC remaining in the supernatant was considered as the yield of fibrillation. The SF was determined via the vacuum filtration of 3 mL of the original cationic MNFCs suspension (ca. 1 wt %) through a cellulose acetate membrane filter with a 0.2 µm pore size. The weight percentage of CC on the filtrate was considered as the soluble fraction. More detailed information regarding the characterization of these cationic MNFCs is available elsewhere [23].

The weight average molecular weight (avgMw) of the cellulose polyelectrolytes was determined by size exclusion chromatography (SEC) in an Agilent 1260 Infinity II High-Temperature GPC System that was equipped with two PL aquagel-OH Mixed-H 8µm (300 × 7.5 mm) columns and a PL aquagel-OH 8µm (50 × 7.5 mm) guard column [23].

### 2.4. LDS Flocculation Tests

To access the performance of the produced cationic celluloses to flocculate PCC, the evolution of the size distribution of the PCC flocs was monitored in a Mastersizer 2000 device (from Malvern Instruments) equipped with the Hydro 2000 module, in a similar way to that proposed by Rasteiro et al. [31,32,35] for the screening of synthetic polyelectrolytes.

Previously to the measurements, a 1 wt % aqueous suspension of PCC was subjected to magnetic stirring for 30 min and ultrasonicated for 15 min at 50 kHz to help disaggregate the PCC particles. The suspension was then maintained under magnetic stirring. For the flocculants tested (CPAM, BEKP and cationic celluloses), 0.1 wt % of aqueous suspensions were prepared and kept under magnetic stirring.

For the flocculation experiment, 15 mL of the PCC suspension was added to the equipment dispersion unit vessel containing 700 mL of distilled water, resulting in a PCC concentration of around 0.02 wt % (a laser-obscuration level of nearly 30% [32]) and a pH of 9. The pump speed was set to 1400 rpm.

In an initial test, after the stabilization of the PCC median size, a certain amount of flocculant (1 mg of flocculant/g of PCC) was added to the measuring vessel every single minute until a maximum of 15 mg of flocculant was reached (15-min test).

Although this procedure is not typical in flocculation studies, and although that one minute between the subsequent additions of flocculant is not enough time for all the flocculation steps (mixing, adsorption, reconformation and aggregation) to occur, it has proven to be a faster and more efficient way of screening those flocculants that offer the best potential. This method was also previously used with synthetic flocculant to access the optimum dosage [32]. Additionally, this strategy is quite common when prescreening flocculants for flocculation in effluent treatment, wherein offline tests with the continuous addition of consecutive doses of the flocculant are conducted (jar tests) [40], in order to optimize the flocculant dosage, the difference being that, in that case, the normal strategy does not involve the simultaneous measurement of the particle size.

For the samples presenting the best performance in the previous test, a second study was conducted to study the evolution in the size of the flocs over time for a fixed concentration of flocculant. In this test, after the addition of the PCC, a certain amount of flocculant (1, 2, 4, 8 or 10 mg/g of PCC) was added to the PCC suspension at once. The evolution of the size of the flocs was measured every minute for 15 min. All the measurements were performed in triplicate. A test with only PCC (no addition of flocculant), wherein the size was monitored for 15 min, was performed for comparison.

The minimum flocculant dosage considered in this study (1 mg/g of PCC) was based on the typical values used in a papermill for the flocculation of mineral fillers. Other authors have tested similar dosages of bioflocculants for the flocculation of distinct minerals. As an example, Sirviö et al. [25] tested dosages of up to 9 mg/g of mineral, while Aguado et al. [15] tested the addition of 20 mg/g of mineral. Higher dosages, of up to 500 mg/g of mineral, were tested by Campano et al. [41].

### 2.5. Mass Fractal Dimension of the PCC Flocs

Besides the mean size and size distribution, LDS also allows us to infer the structure of the formed flocs by means of mass fractal dimensions (d_F_) [31,42,43]. The d_F_ expresses the degree to which primary particles fill the space within the nominal volume occupied by an aggregate; this can be used as an indication of the fluffiness/density of the flocs (Equation (1)):

m(R) ∝ R^d^_F_ (1)

The mass (m) of any fractal aggregate is directly proportional to its radius (R), raised to the exponent, d_F_ [44]. A d_F_ value tending to 1 indicates a more stringy and less dense floc structure, while a value approaching 3 suggests stronger and denser flocs [42].

According to the Rayleigh–Gans–Debye (RGB) theory, the d_F_ can be calculated from the negative slope of the log-log plot of the scattered light intensity versus the scattering wave vector (q) (Equation (2)) [35,44]:

q = (4πn_0_/λ_0_)sin(θ/2) (2)

n_0_ is the refractive index of the dispersant medium (1.33 for water), θ is the scattering angle (between 0.01° and 40.6° for the Mastersizer 2000 equipment) and λ_0_ is the incident light wavelength in vacuo (630 nm).

The validity of the RGB theory is based on the assumption that the elementary units scatter light independently, this being more appropriate for sub-micron spherical particles with low refractive index [43] and applicable when the region of study is much larger than the primary particles and is much smaller than the floc aggregates (Equation (3)) [45].

1/R_agg_ << q << 1/R_part_ (3)

R_agg_ is the radius of the aggregate and R_part_ is the radius of the primary particle.

For systems where the flocculation is very pronounced and the size of the flocs (secondary aggregation resulting from the aggregation of primary flocs) falls out of the applicability range of the RGB theory, the information about the flocs is provided by the scattering exponent (SE) [43,46]. The SE corresponds to the negative slope of the log-log plot of the scattered light intensity, versus q at large length-scales (low diffraction angles).

**Figure 2:**
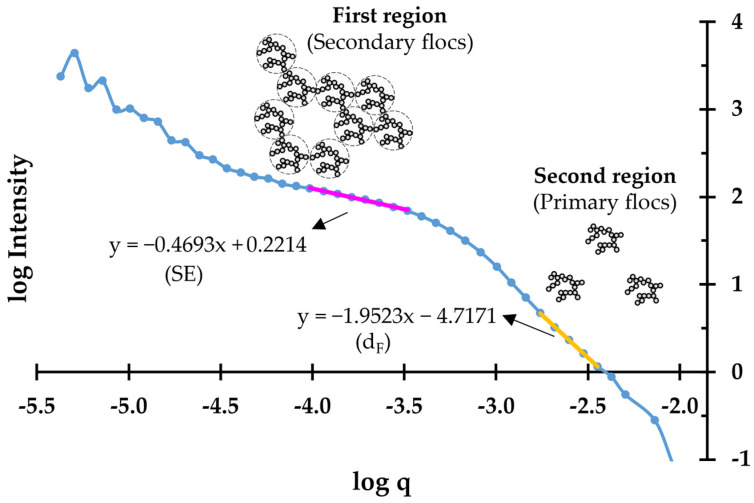
Example of the log-log plot of scattering light intensity versus q for the determination of the scattering exponent, SE (first region), and the fractal dimension, d_F_ (second region).

In short, structural information about the flocs in a certain system can be obtained from the d_F_ for small-length scales (high diffraction angles) and from SE for high-length scales (small diffraction angles), as exemplified in Figure 2, corresponding to the primary and secondary aggregates, respectively.

The structural information (d_F_ and SE) of the flocs that were formed in the second flocculation study (best-performing samples) was obtained by outputting the raw data of the LDS equipment to an external spreadsheet (provided by Malvern Instruments) for the offline analysis of the data and for computing the angle of each detector and the intensity of light at each detector. Making use of Equation (2), the data was further computed into log-log plots of scattered light intensity versus the scattering wave vector (q) (Figure 2), when either d_F_ or SE could be obtained.

### 2.6. Drainability and Retention Tests

Drainability and retention tests were performed with the commercial CPAM and the cationic celluloses that presented the best performance in the LDS flocculation tests.

Before carrying out the drainage tests, suspensions of the different required components were prepared.

The same BEKP (presenting a refining degree of 17° SR) that was used to prepare the cationic celluloses was used for the drainability tests in the form of a 1 wt % solids content suspension. The PCC was prepared as a 1 wt % solids content suspension. The commercial CPAM and the produced cationic celluloses were prepared as 0.1 wt % solutions/suspensions (as described previously for the flocculation tests).

The drainage times were recorded in a dynamic drainage analyzer (DDA, AB Akribi Kemikonsulter, Sweden) for formulations with pulp, filler and retention agent (CPAM or cationic cellulose) [8].

The formulations were prepared in order to obtain sheets with a nominal filler content of 20 wt %. For reference, tests using only pulp and PCC (80 BEKP: 20 PCC) and without a retention agent were performed. Contents of 0.02, 0.2 and 0.4 wt % of cationic cellulose (corresponding to 1, 10 and 20 mg of cationic cellulose per gram of PCC) were tested and compared to samples with the addition of 0.02 wt % of CPAM.

The pulp was disintegrated in the DDA vessel at 1200 rpm for 60 s. The filler, pre-flocculated with the retention agent for 60 s in an external beaker with magnetic stirring (700 rpm), was then added to the pulp suspension inside the equipment vessel, reaching a total solids concentration of 5 g/L and a final volume of 500 mL (extra water was added to the pulp during disintegration to achieve the final desired volume). The final suspension was kept at 800 rpm for 60 s and was subsequently drained. The DDA vessel was equipped with a 60-mesh (250 µm pore size) screen and a vacuum of 30 kPa was maintained in the equipment.

After drainage, the formed wet pads were removed from the forming screen and dried at 105 °C. The dried pads were subsequently calcinated at 525 °C (according to the TAPPI standard, T211) to determine the effective filler content and the corresponding filler retention.

## 3. Results and Discussion

### 3.1. Chemical Analysis

**Figure 3:**
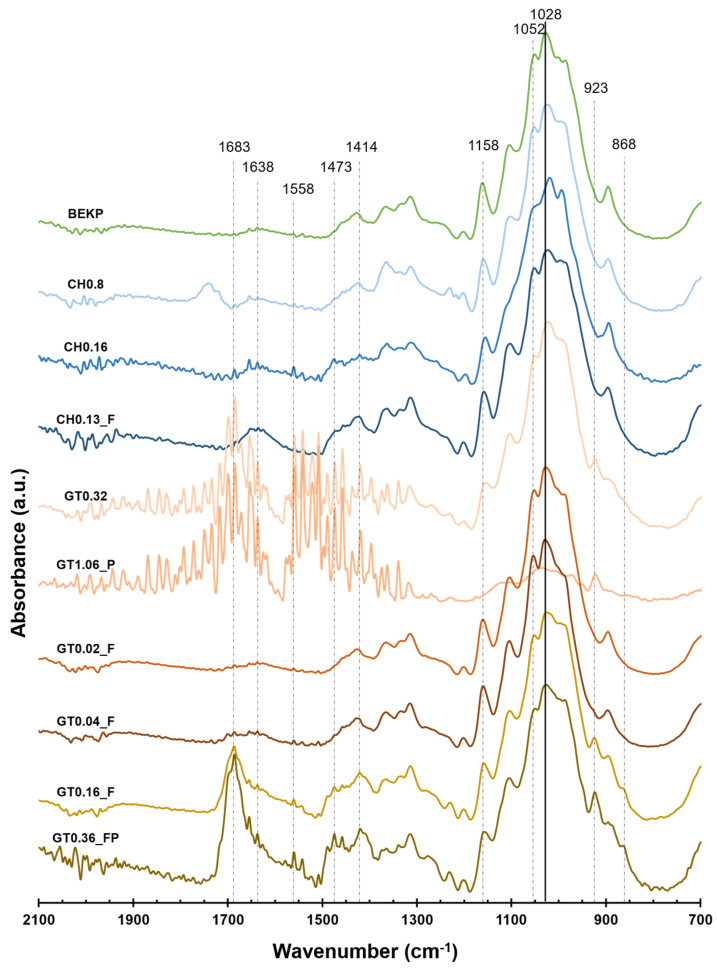
The FTIR-ATR spectra of the original BEKP and celluloses cationized with CHPTAC and GT.

As mentioned before, cationic celluloses with distinct morphologies and levels of cationization were obtained by the incorporation of quaternary ammonium groups into the BEKP structure via direct cationization with CHPTAC or by a two-step reaction (periodate oxidation + Girard’s reagent T) and the submission of some of the samples for treatment in an HPH. The successful cationization was confirmed by the ZP (Table 2) and FTIR (Figure 3) results, and the extent of the cationization was assessed by the determination of the DS and CD (Table 2).

The negative ZP (−23 mV) of BEKP shifted to positive values for all the CCs, with the ZP values ranging between +18 and +35 mV, indicating the successful incorporation of cationic moieties into the cellulose structure.

The chemical modifications induced in the cellulose were analyzed by FTIR-ATR. The FTIR spectra of the original BEKP and CCs, normalized by the highest peak of BEKP (1028 cm^−1^), are presented in Figure 3.

For the samples cationized with CHPTAC, it was possible to observe the appearance of a shoulder at 1473 cm^−1^ related to the stretching vibration of the methyl groups of the quaternary ammonium [48], along with a small peak at 1638 cm^−1^ due to the increased hydrophilicity and consequent water adsorption [49]. The changes are more evident for the sample known as CH0.16, with the highest DS. For the samples subjected to the two-step cationization, a new set of distinct peaks is visible (this is less evident for samples GT0.02_F and GT0.04_F, due to their low DS). The absorbance at 1683 cm^−1^ can be assigned to the stretching of the carbonyl group on the GT structure [50]. The band at 1558 cm^−1^ confirms the formation of the imine bond between DAC and GT [50]. The peaks found at 1473 cm^−1^ and 1414 cm^−1^ are related to the asymmetric and symmetric bending of the methyl groups of GT, respectively [26]. The peak at 923 cm^−1^ can be assigned to the asymmetric stretching of the C–N bond [26], the N–N bond [50,51], or to the stretching vibration of the C–C= bond [52] of the GT groups.

The glucose ring-opening and subsequent oxidation of the hydroxyl groups into aldehyde groups during the periodate oxidation of cellulose can justify the decrease in intensity observed in the bands at 1158 cm^−1^ and 1052 cm^−1^ [26,53]. The appearance of a small peak at 868 cm^−1^ can be associated with the existence of hemiacetal bonds that formed between the remaining aldehyde groups and nearby hydroxyl groups [54].

The content of quaternary ammonium groups introduced into the cellulose structure was quantified in terms of DS and CD (Table 2) via elemental analysis and potentiometric titration, respectively. The samples cationized with CHPTAC presented a DS in the range of 0.08–0.16, while for those prepared by the two-step reaction with GT, a range of 0.02–1.06 was obtained. The CD of the CCs was estimated in the range of 0.23–3.44 mmol/g.

### 3.2. Morphological Analysis

**Figure 4:**
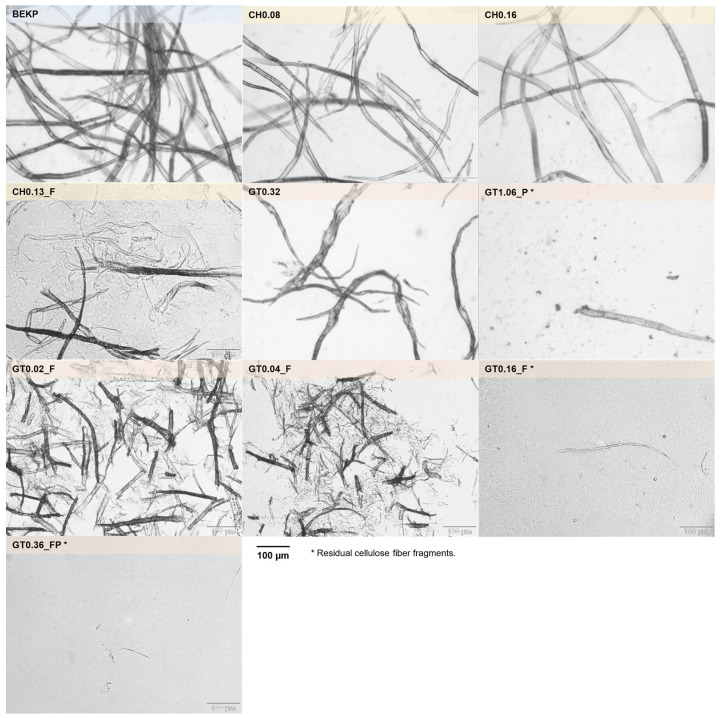
Optical microscopy images of the original BEKP and celluloses cationized with CHPTAC and GT (* Residual cellulose fiber fragments).

Figure 4 displays optical microscopy images of the original BEKP and the produced CCs, all at the same magnification. For the samples CH0.08 and CH0.16, no visible morphological changes are observed in the cellulose fibers, keeping their structure intact and similar to that of the original BEKP. The HPH treatment of the original fibers led to the partial disruption of the cellulose structure, inducing the formation of long micro/nanofibrils, as visible in sample CH0.13_F. 

For the samples obtained by the two-step reaction, sample GT0.32 presents fibers with an increased diameter and the appearance of balloons along the fibers, without the noticeable release of fibrils into the medium, while sample GT1.06_P, reveals that the initial cellulose fibers were almost completely solubilized (with the exception of some small, visible fibers fragments remaining, probably due to some cross-contamination).

The remaining samples (GT0.02_F, GT0.04_F, GT0.16_F and GT0.36_FP) that were obtained after the HPH treatment clearly show the effect of the mechanical treatment. Samples GT0.02_F and GT0.04_F reveal that the cellulose fibers broke down into smaller fiber fragments, along with the liberation of micro/nanofibrils into the medium (which are shorter than those present in sample CH0.13_F). Similarly to sample GT1.06_P, for samples GT0.16_F and GT0.36_FP, only residual small fiber fragments can be seen, with all the cellulose fibers being converted to micro/nanofibrils and then partially or totally solubilized.

The samples treated in the HPH were also characterized in terms of YF and SF to quantify the deconstruction level suffered by the cellulose fibers. Sample CH0.13_F presents a YF of 29% and shows no solubilization of cellulose fibers (SF of 0%) (Table 2). All the samples that were cationized with GT have solubilized cellulose, ranging from 6.1% for GT0.02_F to 99.6% for GT0.36_FP. As for the YF, it increases from 6.4% (sample GT0.02_F) to 100% (sample GT0.36_FP).

For the Mw of the cationic cellulose polyelectrolytes (GT0.36_FP and GT1.06_P) and for the soluble fraction of sample GT0.16_F, a bimodal distribution was obtained (Table 2), with samples GT0.36_FP and GT0.16_F presenting peaks near 1000 and 500 Da and sample GT1.06_P presenting peaks at 2150 and 972 Da.

### 3.3. Performance in the Flocculation Tests

**Figure 5:**
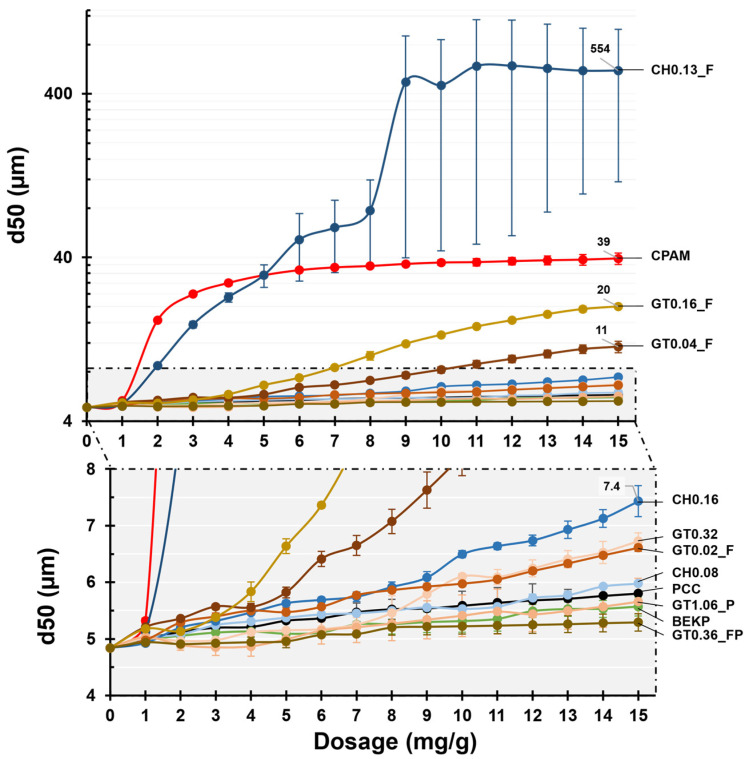
The median floc size as a function of flocculant dosage (the *x*-axis corresponds to both the flocculant dosage and the time of flocculation).

The evolution of the d_50_ of the PCC flocs formed by the contact of the PCC with the retention agent (CPAM or CCs) as a function of the dosage of the retention agent, monitored by LDS, is plotted in Figure 5. 

When no retention agent was added to the PCC suspension (black line), the d_50_ of PCC suffered a small increase from the initial 4.8 µm to 5.8 µm after 15 min, indicating a small natural tendency for aggregation. The reference synthetic commercial retention agent (CPAM) presents a curve distinct from those using CCs. CPAM resulted in the best performance for dosages below 5 mg/g of PCC, also showing the fastest flocculation kinetics up to a dosage of 2 mg/g of PCC (16.5 µm), from which the size of the flocs starts to stabilize, leveling off at ca. 40 µm. These results indicate that a dosage of CPAM above 2 or 3 mg/g of PCC does not bring benefits in terms of flocculation.

With the addition of the original BEKP, no changes were observed in the flocculation of PCC, with the flocs achieving a size of 5.6 µm after the final addition of 15 mg/g of PCC (as with the PCC suspension alone). Similar results were detected for the samples CH0.08, GT1.06_P and GT0.36_FP, with flocs reaching a final size of 6.0, 5.7 and 5.3 µm, respectively. Samples GT0.02_F, GT0.32 and CH0.16 resulted in floc sizes that were slightly bigger than those of the PCC without a retention agent, with the final sizes ranging between 6.6 and 7.4 µm.

However, with samples GT0.04_F and GT0.16_F, an increase in flocculation performance was observed, resulting in a final floc size of 11 and 20 µm, respectively; sample CH0.13_F led to the best flocculation performance, exhibiting an average d_50_ of 554 µm. Contrary to the other samples, the increased tendency of this latter sample to promote PCC flocculation also resulted in a high standard deviation of d_50_, clearly visible after an addition level of 5 mg/g of PCC and is indicative of more heterogeneous flocculation.

Comparing the curves obtained for the distinct cellulose samples and considering their morphological and chemical properties, some major tendencies can be derived. Grouping the samples composed of intact cellulose fibers (BEKP, CH0.08 and CH0.16), it is possible to observe a small increase in flocculation performance with an increasing level of cationization. Sample GT0.32, wherein the cellulose fibers start showing signs of degradation, led to a slight decrease in the average size of the flocs. This behavior is an indication that the cationic charge of the fibers (with a DS of 0.32) is becoming excessive (compared to CH0.16, with a DS of 0.16) and, since the PCC used in this study presents a slightly positive charge, this can lead to charge repulsion. An increase in cationization (GT1.06_P) resulted in the complete absence of flocculation, further strengthening the hypothesis of excessive cationicity.

The positive effect of HPH treatment and the difference between the two distinct cationization methods can be observed by comparing sample CH0.16 with samples GT0.16_F and CH0.13_F. With the fibrillation of the cellulose fibers and the subsequent increase in the available specific surface area, the final d_50_ increased by ca. 3 times for GT0.16_F and ca. 75 times for CH0.13_F. Although both samples present a similar DS and were subjected to the same HPH treatment, they led to very distinct flocculation performances. Such behavior can be explained by the length difference of the obtained fibrils, with sample GT0.16_F presenting a more degraded structure and, even, partially solubilized cellulose, a consequence of the harsher cationization reaction, namely, the sodium periodate oxidation that leads to the opening of the glucose rings, as explained elsewhere [23].

The fibrillated samples obtained by cationization with GT presented an increased flocculation behavior from sample GT0.02_F up to sample GT0.16_F with higher DS. An increase in DS up to 0.36 (GT0.36_FP) resulted in the complete solubilization of the sample and complete loss of the flocculation potential.

**Figure 6:**
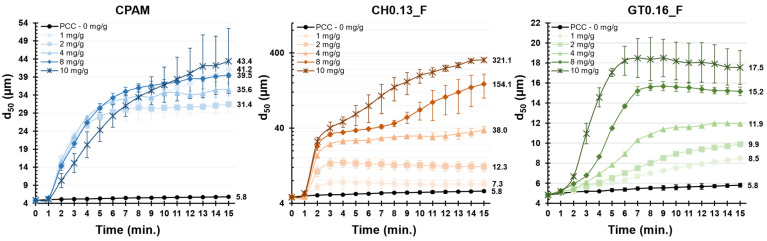
Median flocs size as a function of flocculation time for several concentrations of the best flocculants.

The two best-performing CCs (CH0.13_F and GT0.16_F) and CPAM were subjected to further flocculation tests. The growth of the PCC flocs was recorded over time (15 min) for a fixed dosage of the retention agent (1, 2, 4, 8 and 10 mg/g of PCC), added all at once at 0 min. The obtained flocculation curves are plotted in Figure 6.

The evolution of the size of the flocs obtained with CPAM, for the various dosages tested, shows a very distinct behavior compared to both CCs. Independently of the dosage of CPAM, the flocculation curves are very similar, with the flocs reaching a size of ca. 31 µm for a dosage of 1 mg/g of PCC and a size of ca. 43 µm for the highest dosage of 10 mg/g of PCC. Although the highest dosage ultimately resulted in the largest floc sizes, it is possible to verify a decline in the flocculation rate, at the initial minutes of flocculation (1 to 7 min), compared to the lower dosages. Such behavior can be an indication of an excess of polymer, resulting in slower flocculation kinetics due to the changed diffusion barriers and repulsive forces [55].

With the CCs, the size of the final flocs seems to be very dependent on the initial dosage of polymer. Sample CH0.13_F presented an initial flocculation peak at around the 2-minute mark (similar to CPAM). At a dosage of 1 and 2 mg/g of PCC, the flocculation was slower than with CPAM, whereas, at a dosage of 4 mg/g of PCC or above, the opposite occurred. With an addition of 1 to 4 mg/g of PCC, there was an initial increase in the size of the flocs until the 2-minute point, with the floc sizes stabilizing afterward until the end of the test (7 to 38 µm). For a higher dosage (8 and 10 mg/g of PCC) the flocs continued to increase in size until the end of the test (ca. 154 and 321 µm, respectively).

Sample GT0.16_F showed the slowest flocculation kinetics among the three samples. While both CPAM and CH0.13_F presented an initial burst in flocculation for all the dosages tested, the sample GT 0.16_F presented a slow and steady increase in the size of the flocs at a lower dosage (1 and 2 mg/g of PCC), with the flocculation kinetics increasing for higher dosages. For the tested dosages of 1 to 10 mg/g of PCC, a final floc size of between 8.5 and 17.5 µm was obtained.

In the literature, CPAM of high Mw and low to medium CD have been utilized to promote flocculation via the bridging mechanism (which is typically associated with a fast flocculation rate) [32].

Thus, due to the low CD and long fibrils, bridging is probably the dominant mechanism for sample CH0.13_F. The existence of residual carboxyl groups (ionized at pH 9) in the cellulose fibers’ structure, and their possible attractive interaction with quaternary ammonium groups (and also with the slightly positively charged PCC), may favor the entanglement of the long fibrils between adjacent flocs; this trend may explain the extreme size of the flocs obtained for 8 and 10 mg/g of PCC.

On the other hand, sample GT0.16_F presented slower flocculation kinetics. This reduction in the average size of the flocs, comparatively to CH0.13_F, is a result of the higher level of degradation of the fibrils (shorter fibrils) and the partial solubilization of the sample (SF of 40%).

Observing the various flocculation curves, it is possible to verify that not all the systems were able to achieve a steady state. Nevertheless, since the focus of the work is to explore the potential of cellulose-based flocculants for use in papermaking, and because, in the papermaking process, the available contact time between the flocculant and mineral filler before paper production is lower than 15 min, there is no need to extend the flocculation tests further. Typically, the available time can be even lower than 5 min, and so the most important minutes are the initial minutes of flocculation.

### 3.4. Mass Fractal Dimension of the PCC Flocs

**Figure 7:**
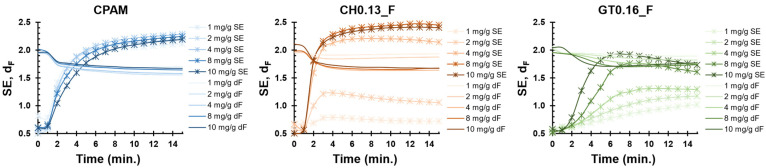
Evolution of the scattering exponent (SE) and mass fractal dimension (d_F_) as a function of flocculation time for several concentrations of the best flocculants.

The d_F_ and SE profiles, calculated from the scattering data for the flocculation tests presented in Figure 6, are plotted in Figure 7. As previously referred to, an analysis of the d_F_ and SE can give some insights regarding the structure (density) of the primary and secondary aggregates, respectively.

In this flocculation system, the primary particles can be seen as the individual smaller PCC particles. For all three samples, the d_F_ started at ca. 2, dropping to ca. 1.7 after 2 min.

Regarding the SE profile, which is more representative of the larger aggregates formed during the flocculation process, at the beginning of the test, as the secondary aggregates started to form, they presented a SE of ca. 0.5 (low density); as time elapsed and flocculation progressed, the secondary aggregates become more compact due to the inclusion of more particles, reaching steady compactness.

When using CPAM, independently of the dosage used, SE increased rapidly in the initial minutes, coinciding with the fast growth of the flocs. After 6 min, the SE slowly stabilized at around 2.2.

The flocs obtained with both CCs became more compact with increasing levels of addition.

For sample CH0.13_F, a dosage of 1 or 2 mg/g of PCC resulted in low SE values (0.7 and 1.1, respectively). A dosage of 4 mg/g of PCC or above produced flocs with a compactness similar to that of those obtained with CPAM (SE of ca. 2.4). As for sample GT0.16_F, at the lowest dosage, flocs that were slightly denser than those obtained with CH0.13_F (SE of 1 versus 0.7) were formed. At a dosage of 2mg/g of PCC, similar densities were obtained for both CCs (SE of ca. 1.1). With a further dosage increase, the flocs became more compact, achieving a maximum SE of ca. 2 at the highest dosage of 10 mg/g of PCC (lower than the 2.2 obtained with CPAM and CH0.13_F).

### 3.5. Drainability and Filler Retention

**Figure 8:**
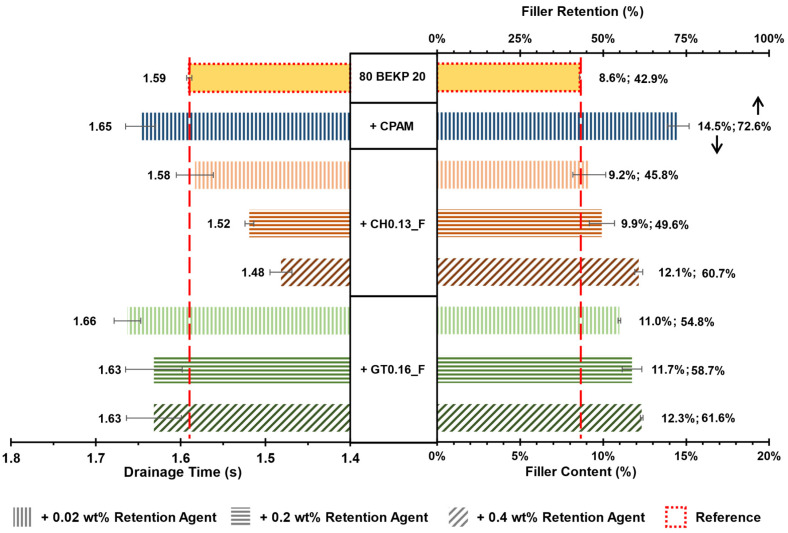
Drainage time, filler content and filler retention as a function of dosage of the best flocculants.

The drainage time and filler content/filler retention results for the pulp suspension containing pre-flocculated PCC are presented in Figure 8. A test with only pulp and PCC (no retention agent) was performed as a reference. Considering the previous flocculation results obtained by LDS, where CPAM demonstrated almost the same performance for all the dosages tested, only the lowest dosage (1 mg/g of PCC or 0.02 wt % addition) was used in the DDA tests. For both CCs (CH0.13_F and GT0.16_F), three levels of addition were tested (1, 10 and 20 mg/g of PCC, equivalent to an addition of 0.02, 0.2 and 0.4 wt %).

For the suspension with only pulp and PCC (reference test), the drainage time was 1.59 s. From the target filler content of 20 wt %, only ca. 43% was retained during drainage (less than half the added amount), resulting in an effective final filler content of 8.6 wt %.

With the addition of CPAM (1 mg/g of PCC), the filler retention increased to ca. 73%, corresponding to a filler content of 14.5 wt % (an increase of ca. 70% versus the reference). As a consequence of the increased retention, an increase in the drainage time to 1.65 s was also observed.

For both CCs, an increase in filler retention was always detected, which retention directly improved with the level of addition. Nevertheless, none of the formulations led to better retention than that of CPAM.

Sample GT0.16_F led to a better filler retention than sample CH0.13_F at 54.8, 58.7 and 61.6% against 45.8, 49.6 and 60.7%, for an addition of 0.02, 0.2 and 0.4 wt %, respectively.

In terms of drainability, sample CH0.13_F, always presented drainage times that were lower than the reference and that decreased with the increase in the addition level, with a reduction of ca. 7% to 1.48 s for the highest dosage. For sample GT0.16_F, the drainage times remained slightly higher than the reference.

The decline in the drainage time observed for sample CH0.13_F when higher dosages were used can be explained by the excessive tendency of this sample to flocculate (as observed by LDS), promoting the formation of large and porous flocs of fibers. These big flocs can result in the heterogeneity of the pad, which leads to regions with fewer fibers that promote preferential drainage channels and a consequent decrease in the drainage time. The existence of these channels may also favor the loss of small PCC flocs that are not properly stuck to the fiber network, which may explain why the structure of sample CH0.13_F, although demonstrating a higher flocculation performance than GT0.16_F in the LDS flocculation tests, did not translate into improved filler retention.

## 4. Conclusions

Several cationic cellulose derivatives with distinct morphologies, namely, fibers, micro- and nanofibrillated celluloses and soluble celluloses, with degrees of substitution ranging between 0.02 and 1.06, were produced by the incorporation of quaternary ammonium groups into the cellulose structure. A direct reaction with CHPTAC or a two-step reaction with Girard’s reagent T was used for cationization. Cationic micro- and nanofibrillated celluloses were produced by subsequent high-pressure homogenization.

The produced samples were evaluated by LDS in terms of their potential to flocculate PCC (in terms of the average size of flocs) and were compared against a commercial CPAM.

The fibrillated celluloses with a DS of 0.13 (CH0.13_F, obtained by the CHPTAC pretreatment) and 0.16 (GT0.16_F, obtained by the Girard T pretreatment) demonstrated the highest flocculation potential, being the best and second-best performing samples, respectively. The flocculation of sample CH0.13_F at dosages above 4 mg/g of PCC resulted in floc sizes greater than those obtained with CPAM. The sample GT0.16_F, independently of the dosage used (1 to 10 mg/g of PCC), always led to flocs smaller than with CPAM added at the lowest dosage (1 mg/g of PCC).

Both cationic samples were further incorporated into pulp and filler formulations and were analyzed in a dynamic drainage analyzer to quantify their effects on filler retention. Compared to the reference formulation of only pulp and PCC (no retention aid), where a filler retention of ca. 43% was obtained, both celluloses were able to increase the levels of retention to ca. 61–62% (for an incorporation of 0.4 wt %), remaining, however, below the 73% achieved with CPAM (for an incorporation of 0.02 wt %).

The cationic celluloses were demonstrated to be a promising new bio-based retention agent, with the potential to replace CPAM as a retention agent in papermaking, although the results of this work indicate the need for further studies in order to achieve performances similar to or better than those of the fossil-based retention agents that are used presently, and with equivalent costs.

## Figures and Tables

**Table 1:** Reaction conditions used for the cationization of BEKP with CHPTAC and GT.

**CHPTAC Method**												
**Sample**	**Cellulose Activation**						**Cellulose Cationization**					
	**C ^1^** ** (wt %)**	**IPA** **(% *v/v*)**	**NaOH/AGU ^2^ (mol/mol)**		**T** **(°C)**	**t** **(h)**	**CHPTAC/AGU ^3^ (mol/mol)**		**T** **(°C)**		**t** **(h)**	
CH0.08	2	-	12		5	1	3		65		8	
CH0.16	5	50	3.4		70	1	2		70		2	
CH0.13_F	3	-	9		20	0.3	6		65		8	
**GT method**												
**Sample**	**Cellulose Oxidation**						**Cellulose Cationization**					
	**C ^1^** ** (wt %)**	**IPA** **(% *v/v*)**	**T** **(°C)**	**t** **(h)**	**SP/AGU ^4^ (mol/mol)**	**Dsa ^5^**	**C ^1^** ** (wt %)**	**AA** **(% *v/v*)**	**IPA** **(% *v/v*)**	**GT/ald.^6^ (mol/mol)**	**T** **(°C)**	**t** **(h)**
GT0.32	2.5	10	70	4	0.50	0.50	5	- ^7^	-	1	70	1.5
GT1.06_P					1.50	1.59		10	60			
GT0.02_F					0.10	0.11			-			
GT0.04_F					0.20	0.19			-			
GT0.16_F					0.55	0.48			-	0.7		
GT0.36_FP					0.65	0.85			-	0.6		

^1^ Pulp consistency; ^2^ molar ratio of NaOH/Anhydroglucose unit; ^3^ molar ratio of CHPTAC/anhydroglucose unit; ^4^ molar ratio of sodium periodate/anhydroglucose unit; ^5^ degree of substitution of aldehyde groups; ^6^ molar ratio of Girard’s reagent T/aldehyde group; ^7^ pH adjusted to 4.5 with dilute HCl.

**Table 2:** Characterization of the original BEKP and celluloses cationized with CHPTAC and GT.

	DS	CD (mmol/g)	ZP (mV) ^1^	YF (%)	SF (%)	avgMw (Da) ^2,3^/PDI ^4^
BEKP	-	0.14 ± 0.01 ^5^	−23 ± 5	-	-	-
CH0.08	0.08 ± 0.02	0.46 ± 0.04	+29 ± 3	-	-	-
CH0.16	0.16 ± 0.00	0.92 ± 0.04	+18 ± 4	-	-	-
CH0.13_F ^6^	0.13 ± 0.01	0.78 ± 0.01	+26 ± 3	29.0 ± 0.4	0.0 ± 0.0	-
GT0.32	0.32 ± 0.01	1.62 ± 0.09	+21 ± 4	-	-	-
GT1.06_P	1.06 ± 0.00	3.44 ± 0.00	+35 ± 2	-	100.0 ± 0.0	2150 ± 12/1.01 ± 0.00 972 ± 8.5/1.02 ± 0.00
GT0.02_F ^6^	0.02 ± 0.01	0.23 ± 0.04	+24 ± 2	6.4 ± 0.6	6.1 ± 1.0	-
GT0.04_F ^6^	0.04 ± 0.01	0.32 ± 0.03	+29 ± 5	11.5 ± 1.0	6.5 ± 1.6	-
GT0.16_F ^6^	0.16 ± 0.01	0.90 ± 0.02	+27 ± 2	98.4 ± 0.5	39.9 ± 1.4	1081 ± 3.5/1.11 ± 0.00 526 ± 0.5/1.02 ± 0.00
GT0.36_FP ^6^	0.36 ± 0.01	1.68 ± 0.04	+30 ± 3	100.0 ± 0.0	99.6 ± 0.4	1025 ± 43/1.06 ± 0.02 543 ± 4.5/1.01 ± 0.00

^1^ Determined at ca. pH 7; ^2^ bimodal distributions; ^3^ measured for the soluble fraction of the sample; ^4^ polydispersity index; ^5^ carboxyl content of the initial BEKP, determined by conductometric titration [47]; ^6^ Data from [23].

## Data Availability

Not applicable.
